# Unusual Pathogen in a Patient With Peritoneal Dialysis-Related Peritonitis: A Case Report

**DOI:** 10.7759/cureus.23948

**Published:** 2022-04-08

**Authors:** Alya Z Aboulhosn, Rebecca A Takele, Paul F Laflam

**Affiliations:** 1 Nephrology, Edward Via College of Osteopathic Medicine, Blacksburg, USA; 2 Nephrology, Carilion New River Valley Medical Center, Christiansburg, USA

**Keywords:** peritoneal dialysis-related peritonitis, kidney failure, peritonitis, pasteurella multocida, peritoneal dialysis, dialysis

## Abstract

Peritoneal dialysis (PD) is an effective modality for renal replacement therapy. A serious complication that can arise from PD is peritonitis. Over the last few decades, there have been cases of PD-related peritonitis secondary to *Pasteurella multocida *infections*.* We present the case of a 44-year-old female who presented to the emergency department with a one-day history of abdominal pain and cloudy peritoneal fluid on evaluation. Along with her physical examination findings, laboratory results of the peritoneal fluid demonstrated elevated white blood cells and neutrophils, characteristic of peritonitis. Ultimately, the culture results were positive for *P. multocida.* Although *P. multocida *is not the most common cause of peritonitis, it is a common cause in PD patients who have domesticated animals. With two out of three people being pet owners and the increased number of people on home therapies such as PD for kidney failure, it is important to educate patients about the proper precautions and techniques to prevent peritonitis and its associated complications. Additionally, proper antibiotic management should be implemented for patients with an increased risk of infection.

## Introduction

Renal replacement therapy modalities for kidney failure include in-center hemodialysis (HD) and home dialysis therapies such as peritoneal dialysis (PD), daily home HD, nocturnal home HD, and standard home HD. The Advancing American Kidney Health initiative was created by executive order with a goal of having 80% of kidney failure patients treated with home dialysis or kidney transplant by 2025 [1]. Approximately 15% of all dialysis patients are on PD, while the other 85% are on HD [2]. Current studies show that in the first few years, patients on PD have better improved residual renal functions and mortality rates than those on HD. However, PD is not without inherent risks and complications, such as peritonitis, weight gain, and hernias [2].

Peritonitis is known to be a major contributor to increased morbidity and hospitalization among those receiving PD [3]. The most commonly associated peritonitis-causing organisms are gram-positive bacteria, specifically *Staphylococcus aureus*, and coagulase-negative *Staphylococcus *species, including *Staphylococcus epidermidis*. However, over the last three decades, the number of *Pasteurella multocida*-associated peritonitis cases in PD patients has been increasing. As a gram-negative coccobacillus zoonotic organism, *P. multocida* is transmitted to humans from the normal oral cavity flora of domestic household animals, most commonly cats and dogs [4].

In this case, the patient presented with *P. multocida*-induced peritonitis which is believed to have resulted from improper precautions taken with her cats while on her PD at night. There have been 44 similarly reported *P. multocida*-induced peritonitis cases in patients receiving PD, all within the last three decades [5].

## Case presentation

In December 2021, a 44-year-old White female with a medical history of kidney failure on PD, type 2 diabetes with diabetic nephropathy and neuropathy, and hemochromatosis presented to the emergency department for abdominal pain ongoing for one day. On presentation, she stated that her abdominal pain began after sitting uncomfortably on a salon chair for a prolonged period. She also reported intermittent nausea but denied any vomiting. Her surgical history was significant for prior cesarean, peritoneal catheter placement, and a recent arteriovenous fistula placement.

On admission, the patient was afebrile with a blood pressure of 143/65 mmHg, heart rate of 94 beats per minute, respiratory rate of 16 breaths per minute, and oxygenation saturation of 97% on room air. On physical examination, she had diffuse abdominal tenderness without guarding or rebound and negative Murphy, Lloyd, and Rovsing signs. A PD catheter was in place, and no erythema, warmth, or tenderness was noted at the site of the catheter. The remainder of her physical examination was unremarkable.

Labs and imaging were ordered. Her complete blood count was acutely significant for mild leukocytosis with a white blood cell count of 12.4 × 10^9^ L. The comprehensive metabolic panel was within the patient’s baseline range. Computed tomography (CT) scan of the abdomen and pelvis without contrast revealed no definite acute abnormality. The patient was hemodynamically stable and subsequently discharged home with outpatient follow-up in the clinic.

However, the following day after being discharged, her abdominal pain worsened, and she developed non-bloody, watery diarrhea. She also noticed that the fluid from her PD was dark and had sediment. She spoke with the on-call dialysis nurse who referred her back to the emergency department for further management.

During her second visit to the emergency department, a repeat complete blood count revealed an increase in her white blood count from 12.4 × 10^9^ L to 13.6 × 10^9^ L. Lactate and procalcitonin levels were both normal. A sample of her peritoneal fluid was collected for fluid count, cell differential, and culture. The peritoneal fluid analysis showed yellow, hazy fluid with 4,320/mm^3^ leukocytes and 81% neutrophils (Table 1).

**Table 1:** The patient’s peritoneal fluid cell count and differential results.

Specimen	Peritoneal fluid
Color	Yellow
Appearance	Hazy
White blood cell count, fluid	4,320/mm^3^
Neutrophils	81%
Lymphocytes	11%
Mononuclear cells	6%
Eosinophils	2%

While awaiting culture results, she was managed on vancomycin and cefepime intravenously to cover both gram-positive and negative organisms. She was admitted to the hospital for peritonitis secondary to PD. She received one treatment of continuous renal replacement therapy through her arteriovenous fistula and was managed with HD.

Four days later, culture results were positive for 3+ *P. multocida* and sensitive to ceftazidime and imipenem (Table 2). Her antibiotic regimen was switched to six days of ceftazidime to be given during dialysis treatment. The patient was discharged home after resolution of her leukocytosis, response to HD, and return to her baseline status. During her outpatient HD treatment, the patient received cefepime instead of ceftazidime as the antibiotic was not available.

**Table 2:** Culture and gram stain results for the patient’s peritoneal fluid.

Specimen	Peritoneal dialysate
Gram stain	4+ Polymorphonuclear leukocytes, 3+ mixed bacterial flora
Culture result	3+ *Pasteurella multocida*

During the patient’s hospital stay, she reported that she has four cats at home. She denied keeping them away from her PD machine and had intimate contact with them while receiving her PD. Prior to this, the patient had not had PD-related peritonitis.

## Discussion

The first case of *P. multocida*-related PD peritonitis was reported in 1987 in a patient who acquired the infection from a household cat [5]. Since then, there have been an increasing number of peritonitis cases secondary to *Pasteurella* infections acquired from household pets. Possible sources of infections with *Pasteurella *include intimate contact with cats during treatment, cat biting or playing with the tubing and equipment, and generally having a cat near the dialysis machine [6]. To reduce the risk of contamination, patients should prevent their cats from having any contact with the dialysis machine and equipment. For patients with recurrent peritonitis secondary to *Pasteurella *or when there has been obvious contamination from a cat, such as in the case of tube biting, prophylactic antibiotics should be considered.

Early aggressive usage of antibiotics is associated with treatment success of peritonitis. In most cases, empiric treatment includes intraperitoneal antibiotics. For empiric antibiotic coverage, gram-positive bacterial infections are treated with cefazolin or vancomycin and gram-negative infections are treated with a third-generation cephalosporin or an aminoglycoside [4]. Meropenem is added in cases of severe sepsis to provide broad-spectrum coverage. A generally suggested class of antibiotics includes penicillin, amoxicillin, fluoroquinolone, and third-generation cephalosporins [7]. In the case of our patient, she was empirically given vancomycin and cefepime intravenously to cover gram-positive and gram-negative organisms. The antibiotics were not given intraperitoneally because of her PD catheter contamination. Her culture results indicated that she was sensitive to a third-generation cephalosporin.

A unique aspect of this case is the clinical presentation. Patients with peritonitis typically are hemodynamically unstable, present with acute abdominal pain and distention, and on the physical examination, have abdominal rigidity, rebound, and guarding [8]. While our patient did present with acute abdominal pain, she was hemodynamically stable and only had mild tenderness without any rebound, guarding, or rigidity on examination.

## Conclusions

This case report highlights a serious complication of PD caused by an unusual organism. Peritonitis is a major complication contributing to mortality and morbidity in dialysis patients. While to date including our patient there have only been 45 reported cases of PD-related peritonitis secondary to *P. multocida*, it is an important component of diagnostic consideration. Considering the Advancing American Kidney Health initiative goal to have most kidney failure patients on home dialysis, patient education and proper home dialysis management are more important than ever to prevent infections.
